# Molecular Mapping of Quantitative Trait Loci for Fusarium Head Blight Resistance in the Brazilian Spring Wheat Cultivar “Surpresa”

**DOI:** 10.3389/fpls.2021.778472

**Published:** 2022-01-24

**Authors:** Bikash Poudel, Joseph Mullins, Krishna D. Puri, Yueqiang Leng, Anil Karmacharya, Yuan Liu, Justin Hegstad, Xuehui Li, Shaobin Zhong

**Affiliations:** ^1^Department of Plant Pathology, North Dakota State University, Fargo, ND, United States; ^2^Department of Plant Sciences, North Dakota State University, Fargo, ND, United States

**Keywords:** Fusarium head blight, QTL, genotyping-by-sequencing, deoxynivalenol, Surpresa, common wheat (*Titicum aestivum* L.)

## Abstract

Fusarium head blight (FHB) is a devastating disease in wheat. The use of resistant germplasm from diverse sources can significantly improve resistance to the disease. “Surpresa” is a Brazilian spring wheat cultivar with moderate FHB resistance, different from currently used sources. In this study, we aimed to identify and map the genetic loci for FHB resistance in Surpresa. A mapping population consisting of 187 recombinant inbred lines (RILs) was developed from a cross between Surpresa and a susceptible spring wheat cultivar, “Wheaton.” The population was evaluated for FHB by the point-inoculation method in three greenhouse experiments and four field trials between 2016 and 2018. Mean disease severity for Surpresa and Wheaton was 41.2 and 84.9% across the 3 years of experiments, ranging from 30.3 to 59.1% and 74.3 to 91.4%, respectively. The mean FHB severity of the NILs was 57%, with an overall range from 7 to 100%, suggesting transgressive segregation in the population. The population was genotyped using a two-enzyme genotyping-by-sequencing approach, and a genetic map was constructed with 5,431 single nucleotide polymorphism (SNP) markers. Four QTL for type II resistance were detected on chromosomes 3A, 5A, 6A, and 7A, explaining 10.4–14.4% of the total phenotypic variation. The largest effect QTL was mapped on chromosome 7A and explained 14.4% of the phenotypic variation; however, it co-localized with a QTL governing the days to anthesis trait. A QTL for mycotoxin accumulation was also detected on chromosome 1B, explaining 18.8% of the total phenotypic variation. The QTL for FHB resistance identified in the study may diversify the FHB resistance gene pool and increase overall resistance to the disease in wheat.

## Introduction

Fusarium head blight (FHB) is a destructive disease of wheat worldwide. It is primarily caused by the fungus *Fusarium graminearum* in North America and can significantly reduce grain yield and quality (McMullen et al., 2012; Del Ponte et al., 2017). Severe outbreaks of FHB occur when warm and moist conditions persist at wheat anthesis and result in light-weighted, shriveled, and chalky white/pink grains referred to as “tombstones.” Up to 74% reductions in grain yield due to FHB in cereal crops were estimated based on natural disease epidemics, fungicide trials, and artificial inoculation studies (Wegulo et al., 2015). Besides yield losses, grains can be contaminated with deoxynivalenol (DON) produced by the disease, further restricting their end-use. These implications lead to a higher risk for growers, who may adopt more costly management practices or switch to less risky crops (Dahl and Wilson, 2018). Therefore, an integrated approach that incorporates genetic resistance, fungicide application, and agronomic practices is required to minimize losses to the disease.

Host resistance to FHB is a complex quantitative trait usually governed by small-effect quantitative trait loci (QTL) and is strongly affected by environmental conditions (Steiner et al., 2017). No immunity to FHB has been discovered so far, although sources with some levels of genetic resistance have been identified through extensive germplasm evaluations. Five types of host resistances to FHB have been described: type I (resistance to initial infection), type II (resistance to fungal spread within spike), type III (resistance to toxin accumulation or ability to degrade the toxin), type IV (resistance to kernel infection), and type V (tolerance to yield loss) (Schroeder and Christensen, 1963; Mesterházy, 1995; Mesterházy et al., 1999). However, only type II resistance has been extensively characterized and used in breeding programs owing to its stability and simplicity in assessment. Previous studies indicated that some morphological and phenological traits are involved in FHB resistance through modulating the extent of FHB infection and DON accumulation (Mesterházy, 1995; He et al., 2016). Plant height (PH) and the period of anther retention (AR) after anthesis are primarily shown to play a significant role in FHB resistance (Lu et al., 2013; Steiner et al., 2017). In general, shorter plants are observed to show more severe FHB epidemics (Steiner et al., 2017).

Since the first report of FHB resistance QTL in 1999, over 500 QTL conferring FHB resistance, located on all 21 chromosomes, have been reported (Buerstmayr et al., 2020). Genetic variation in wheat gene pools from diverse geographic regions has been a valuable resource to detect FHB resistance and create locally adapted cultivars with elevated resistance to FHB (Buerstmayr et al., 2014). “Sumai3,” a Chinese spring wheat cultivar, is by far the best source of FHB resistance (Zhu et al., 2019). *Fhb1* is one major QTL identified in Sumai3, which mainly confers type II resistance (Bai and Shaner, 1994; Buerstmayr et al., 2009). Using a map-based cloning approach, Rawat et al. (2016) identified a pore-forming toxin-like (*PFT*) gene as a potential candidate conferring the resistance to FHB at the *Fhb1* locus. Further studies indicated that the *PFT* gene exists in both resistant and susceptible wheat genotypes in the 348 wheat accessions analyzed (He et al., 2018). Two most recent studies revealed that a mutation of the histidine-rich calcium-binding gene “*His*” (syn: *TaHRC*) confers FHB resistance at the *Fhb1* locus (Li et al., 2019; Su et al., 2019). However, the role of mutated *TaHRC* in FHB resistance is still not very clear (Lagudah and Krattinger, 2019; Li et al., 2019; Su et al., 2019). Six other QTL besides *Fhb1* have been formally assigned a gene name: *Fhb2*, *Fhb4*, and *Fhb5* derived from wheat, and *Fhb3*, *Fhb6*, and *Fhb7* derived from wheat-alien species (Bai et al., 2018). Several QTL have been identified and showed additive effects allowing gene pyramiding into locally adapted cultivars to achieve a high level of FHB resistance (Kolb et al., 2001; Rudd et al., 2001; Salameh et al., 2011; Clark et al., 2016; Bai et al., 2018; Zhao et al., 2018a,b). When the QTL effects are large enough, substantially enhanced FHB resistance can be readily achieved with marker-assisted selection (Wilde et al., 2007). Sources of FHB resistance used in current wheat breeding programs can be traced back to only a few parents, including Sumai3 and its derivatives (Bai and Shaner, 1994; Buerstmayr et al., 2009; Chu et al., 2011). However, using only one or a few sources of resistance over large crop production areas poses vulnerability to resistance breakdown and severe disease epidemics. Therefore, QTL analysis on diverse resources is essential to enhance FHB resistance in wheat. On the other hand, FHB resistance detected in locally adapted cultivars may be controlled by multiple genes with minor effects and largely unknown genetics, limiting its use in wheat breeding programs (Clark et al., 2016).

“Surpresa” (PI 185843) is a Brazilian spring wheat cultivar previously identified as having moderate resistance to FHB and DON accumulation (Zhang et al., 2008). It was developed by Dr. Iwar Beckman, the father of Brazilian wheat, from the cross made between “Alfredo Chaves-6-21” and “Polyssu” to withstand aluminum toxicity in the Brazilian acid soil problem (Rajaram et al., 1988). Before Sumai3 was utilized in wheat breeding programs in the Americas, cultivar Frontana from Brazil was extensively used as the FHB resistance source (Rudd et al., 2001; Zhu et al., 2019). Frontana primarily confers type I FHB resistance with some type II and type III FHB resistances (Steiner et al., 2004; Yabwalo et al., 2011; Ágnes et al., 2014). Considering the shared ancestry and origin of Frontana and Surpresa, it would be interesting to decipher the genetic basis of FHB resistance in Surpresa. Therefore, the objective of our study was to identify novel QTL for resistance to FHB in Surpresa and determine whether Surpresa and Frontana share QTL for FHB resistance.

## Materials and Methods

### Plant Materials

A bi-parental mapping population consisting of 187 recombinant inbred lines (RILs) (F_2:7_) was developed from the cross between Surpresa (PI 185843) and the FHB-susceptible spring wheat cultivar Wheaton (PI 469271) using the single-seed descent method. Alsen (PI 615543), having a known *Fhb1* locus, was used as a resistant check in all experiments. ND2710, Grandin, and Wheaton were also included as checks in the field disease phenotyping experiments.

### Phenotypic Evaluation

The RILs and parents, together with the checks, were evaluated for reaction to FHB and related agronomic traits in both greenhouse and field experiments between 2016 and 2018. Greenhouse evaluations were conducted in three growing seasons: fall of 2016 and 2017, and winter of 2018. In each greenhouse experiment, the RILs and parents were grown in a 6-inch clay pot with three plants per plot filled with potting mix (Pro-mix biofungicide; Premier Tech Horticulture, Quakertown, PA) and supplemented with slow-release fertilizer (Osmocote Plus 15-9-12 N-P-K plus minors; Everris Inc., Dublin, OH) after planting. The pots were arranged on greenhouse benches in a randomized complete block design (RCBD) with three replications (pots) per line. The greenhouse was supplemented with artificial light for a 14-h photoperiod, with the temperature maintained between 20 and 22°C during the early crop growth period (before anthesis). The inoculum at a concentration of 100,000 spores/mL was prepared by mixing equal numbers of spores from four pathogenic isolates of *F*. *graminearum* collected from North Dakota (two isolates producing 3ADON and two isolates producing 15ADON) (Puri and Zhong, 2010). FHB inoculations were performed at Zadoks growth stage 65 when the plants are at anthesis (Zadoks et al., 1974) using the single-spikelet inoculation method described by Stack et al. (2002), by injecting 10 μL of the spore suspension into a floret in the central spikelet of spikes using a syringe (10 mL BD syringe, Becton Dickinson & Co., NJ) fitted with a needle (26G^1/2^ Precision Glide^^®^^ Needle, Beckton Dickinson & Co., NJ). Eight to ten spikes from each pot were inoculated. The inoculated spikes were lightly misted and then covered with a 5-inch transparent polyethylene bag for 48 h to keep high humidity. The inoculated plants were maintained at 22–24°C in the greenhouse to ensure proper disease development.

Field evaluations were performed in the FHB nursery located in Fargo, North Dakota, in three summer seasons (2016, 2017, and 2018). In 2016, the RIL population and parents along with checks were planted in hill plots arranged as a randomized complete block design with two replications per line. Since the number of spikes per hill plot for FHB inoculation was low in the 2016 summer season, in 2017 we planted the mapping population in short rows of 6 feet, instead of hill-plots, with one replication per line. In 2018, planting and experiment were the same as in 2016, except that four replications were planted per line. In the 2016 and 2018 field experiments, 10–15 seeds were planted per hill plot, and 4–10 spikes in a hill plot were inoculated. In the 2017 field experiment, 30–40 seeds were planted in each short row, and 20–25 spikes from each row (one row per line) were inoculated. The point-inoculation was done as described above for the greenhouse experiments. The overhead misting was set up to run for 5 min after inoculation and then for 5 min in 3 h intervals for 12 h daily during the night (6:00 p.m. to 6:00 a.m.), until 14 days after the latest maturing lines were inoculated.

In 2018, we also assessed FHB resistance of the mapping population along with parents and checks using the corn-spawn inoculation as described by Chu et al. (2011), and three replications of hill plots per line were planted in the Fargo location. To prepare the corn-spawn inoculum, pre-soaked corn was autoclaved in aluminum foil pan (Full steam deep; Western Plastics, Inc., Calhoun, GA) with lids, infected with twenty pathogenic isolates of *F. graminearum* (ten isolates producing 3ADON and ten isolates producing 15ADON), and set aside for 2 weeks. To assure proper ascospore production and uniform disease pressure, the infested corn kernels were applied to the nurseries at a rate of approximately 0.20 kg/m^2^ starting at the jointing stage (Feeke‘s growth stage 5) of wheat, and repeated every 2 weeks until all wheat lines completed anthesis (Feekes growth stage 10.5). During the inoculation period, overhead misting was run overnight for 10 s every hour to ensure high humidity for uniform disease development. Fifteen to twenty heads/hill were rated for FHB severity at 21 days after flowering.

FHB severity (proportion of symptomatic spikelets in a spike) was assessed 21 days post-inoculation in all greenhouse and field experiments. A modified Horsfall-Barrett disease rating scale with nine infection categories to reflect 0, 7, 14, 21, 33, 50, 67, 80, and 100% of disease severity based on visual assessment was used (Stack and McMullen, 1998). The disease severity of each replication was calculated by taking the average severities of all the inoculated spikes in a hill plot/short row.

GH16P, GH17P, and GH18P were used to represent the greenhouse experiments conducted in 2016, 2017, and 2018, respectively. FAR16P, FAR17P, and FAR18P were used to indicate the field experiments performed at the Fargo location in 2016, 2017, and 2018, respectively, with the point-inoculation method, while FAR18C was used for the corn-spawn inoculated experiment conducted at the Fargo location in 2018.

DON content was assessed for grain samples of each line harvested from three FHB-inoculated experiments in 2018: GH18P, FAR18P, and FAR18C. The three DON testing experiments were designated as GH18P-DON, FAR18P-DON, and FAR18C-DON, respectively. The inoculated heads of each line from point-inoculated experiments (GH18P and FAR18P) were harvested at maturity, combined from all replicates, threshed carefully to keep all the seeds, and ground into fine powder. In case of corn-spawn inoculated field experiment (FAR18C), only infected heads were harvested to assess DON accumulation per line. Fine powdered grain sample from each line from each experiment was submitted for DON analysis to the United States Wheat and Barley Scab Initiative (USWBSI) supported laboratory at North Dakota State University.

To determine the role of morphological and phenological traits of Surpresa in FHB resistance, days to anthesis (DA) and plant height (PH) were recorded. DA was measured as the number of days from planting to Zadoks growth stage 65 when the plants are at anthesis (Zadoks et al., 1974). DA was recorded for all the inoculated spikes in each plant in all greenhouse and field experiments between 2016 and 2018. PH (in inches) was measured from the soil surface to the tip of a spike (excluding awns) from greenhouse and field experiments conducted in 2018 only. An average plant height representative of the biological replicates was recorded.

### DNA Extraction and Genotyping-By-Sequencing

Leaf samples from the parents and mapping population were collected at the 2–3 leaf stage and placed in 96-deep well plates, freeze-dried, and ground using QIAGEN TissueLyser (85300; QIAGEN). Genomic DNA was extracted following a protocol slightly modified from Tai and Tanksley (1990). The extracted DNA was then quantified with a Quant-iT PicoGreen assay kit (P7589; Thermo Fisher Scientific) and subsequently used for GBS-library preparation.

GBS library was prepared following the protocol described in Liu et al. (2019). In brief, 200 ng of the genomic DNA sample was digested with *Pst*I and *Mse*I and ligated with a common and a unique barcoded adapter. Then, equal volumes of the ligation product for each sample were pooled into a 5-mL tube, purified with QIAquick PCR purification kit (28104; QIAGEN), and amplified by PCR. Each PCR reaction was performed in a total volume of 200 μL with 2X Taq Master Mix (M0270L; New England BioLabs^^®^^ Inc.), two primers (5 nmol each), and 50 ng/μL genomic DNA for each sample. PCR amplification was performed with denaturation at 98°C for 10 s followed by 18 cycles of annealing at 65°C for 30 s, and finally 30 s extension at 72°C. The PCR product was cleaned up again using a QIAquick PCR purification kit. The GBS library was then sequenced on an Illumina HiSeq 2500 to generate single-end, 100-bp reads at the Genomic Sequencing and Analysis Facility at the University of Texas Southwestern Medical Center at Dallas, Texas. GBS data were then analyzed for SNPs using the TASSEL-GBS pipeline (Glaubitz et al., 2014) with the *Triticum aestivum* IWGSC RefSeq v1.0 as the reference genome (IWGSC, 2018). SNP markers were filtered for an individual read depth greater than 1, minor allele frequency greater than 0.05, and missing data less than 30% to yield 5,681 polymorphic SNP markers.

### Statistical Analysis, Linkage Map Construction, and Quantitative Trait Loci Analysis

The distribution of phenotypic traits assessed in all experiments was tested for normal distribution using the Shapiro-Wilk test, and homogeneity of variances was verified using the Levene’s test (“car” package) in RStudio version 1.1.453 (Fox and Weisberg, 2019; R Core Team, 2020). Type III analysis of variance (ANOVA) for disease severity was calculated with Satterthwaite’s method for each environment using linear mixed effect model in “lmerTest” package (Kuznetsova et al., 2017) in RStudio version 1.1.453. Correlation coefficients between disease severity and DON accumulation were calculated using Spearman’s correlation (a rank-order correlation) as it applies to measure the relationship between two continuous random variables without assuming a normal distribution of variables. Broad-sense heritability, defined as H^2^ = V_*G*_/V_*P*_, for each trait was calculated by restricted maximum likelihood (REML) method in RStudio using the “Sommer” package (Covarrubias-Pazaran, 2018). Heritability coefficients were estimated from the variance components with the equation


H=2V(V+GV/GxYy+V/Eyr)G/,

where V_*G*_ is genotypic variance, V_*GxY*_ is the genotype-by-year interaction variance, V_*E*_ is the residual variance, y is the number of years, and r is the number of replications.

The SNP markers generated from the GBS were evaluated for distorted segregation and missing values. SNPs with >30% missing values were excluded from linkage mapping. A genetic linkage map with GBS-SNP markers was then constructed using the Kosambi mapping function (Kosambi, 1943) and the “egression” mapping algorithm in JoinMap^^®^^ version 5.0 (van Ooijen, 2018). The minimum logarithm of odds (LOD) threshold of 3 was used to determine linkage groups. The long (L) and short (S) arms of each chromosome were identified based on the physical location of centromeres published in ChiP-seq data for CENH3 (Guo et al., 2016).

Seven phenotypic datasets for FHB severity from GH16P, GH17P, GH18P, FAR16P, FAR17P, FAR18P, and FAR18C and three for DON accumulation from GH18P-DON, FAR18P-DON, and FAR18C-DON were analyzed individually for QTL mapping. The QTL analysis on PH and DA from each experiment was also performed individually. A significantly associated QTL was determined using Composite Interval Mapping (CIM) (Zeng, 1994) in QGene v.4.4 (Joehanes and Nelson, 2008). LOD threshold for claiming significant QTL at *P* < 0.05 was determined by performing 1,000 permutation tests (Churchill and Doerge, 1994).

## Results

### Phenotypic Variation in Fusarium Head Blight and Trait Correlations Among Recombinant Inbred Lines and Parents

**FIGURE 1:**
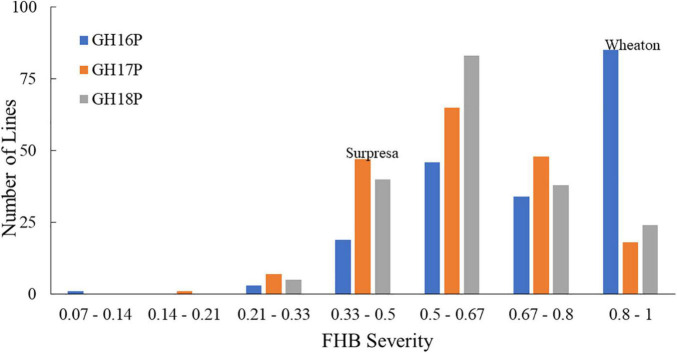
Frequency distributions of FHB severity in the Wheaton/Surpresa RILs across greenhouse experiments. GH16P, GH17P, and GH18P represent the experiments conducted in greenhouse in 2016, 2017, and 2018, respectively, using the point-inoculation method. FHB severity (proportion of symptomatic spikelets in a spike) was assessed and calculated as described by Stack and McMullen (1998).

**FIGURE 2:**
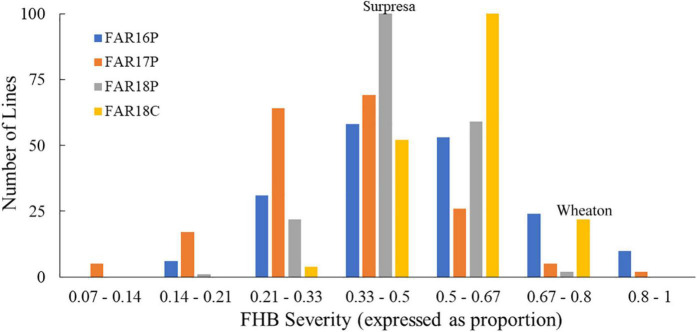
Frequency distributions of FHB severity in the Wheaton/Surpresa RILs across field experiments. FAR16P, FAR17P, and FAR18P indicate the field experiments performed in 2016, 2017, and 2018, respectively, at the Fargo location with the point-inoculation method, while FAR18C was used to represent the corn-spawn inoculated experiment conducted at the Fargo location in 2018. FHB severity (proportion of symptomatic spikelets in a spike) was assessed and calculated as described by Stack and McMullen (1998).

**FIGURE 3:**
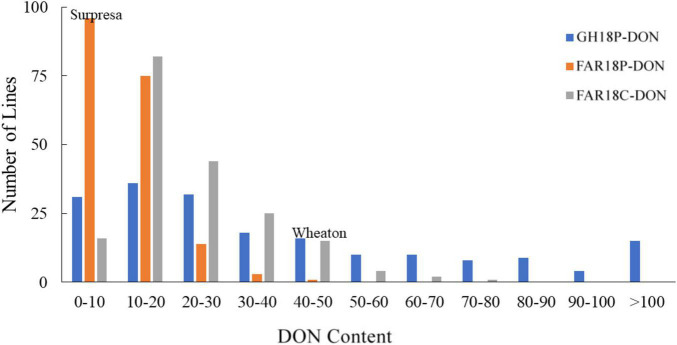
Frequency distributions of deoxynivalenol (DON) content in the Wheaton/Surpresa RILs. DON content (expressed as parts per million, ppm) was assessed for grain samples of each line harvested from three FHB-inoculated experiments in 2018: GH18P, FAR18P, and FAR18C. The three DON testing experiments were designated as GH18P-DON, FAR18P-DON, and FAR18C-DON, respectively.

The FHB severity of the two parents differed significantly (*P* < 0.05), with Surpresa exhibiting moderate resistance while Wheaton was very susceptible in all experiments. Alsen and ND2710, known to possess the *Fhb1* gene derived from Sumai3, showed consistently higher levels of FHB resistance than Surpresa across all experiments. The phenotypic traits and broad-sense heritability for the RILs and the parents are presented in Table 1. Distribution of disease severity and DON accumulation was continuous in all experiments (Figures 1–3), indicating quantitative inheritance of FHB resistance. Disease severities in greenhouse experiments, overall, were higher than in field experiments.

**TABLE 1:** Phenotypes and broad-sense heritability of FHB related traits in Wheaton/Surpresa RILs and parents.

Trait	Experiment	Parents		RILs		
		Surpresa	Wheaton	Mean ± SD	Range	H^2^
FHB Severity	GH16P	na	0.86	0.73 ± 0.24	0.13—1.00	0.64
	GH17P	0.40	0.89	0.60 ± 0.20	0.17—0.95	
	GH18P	0.36	0.91	0.61 ± 0.19	0.22—0.97	
	FAR16P	0.28	0.86	0.50 ± 0.23	0.14—0.97	0.47
	FAR17P	0.59	0.85	0.37 ± 0.15	0.10—0.84	
	FAR18P	0.30	0.66	0.46 ± 0.17	0.19—0.71	
	FAR18C	0.35	0.76	0.55 ± 0.16	0.31—0.76	
DON Content (ppm)	GH18P-DON	7.35	47.10	37.45 ± 30.81	0.33—202.4	–
	FAR18P-DON	3.40	5.90	11.42 ± 6.78	1.00—49.90	
	FAR18C-DON	10.30	39.80	23.30 ± 12.48	6.80—72.10	
Days to anthesis (DA)	GH16P	na	69.00	54.06 ± 5.43	45.00—76.75	0.80
	GH17P	81.00	73.00	54.99 ± 5.84	44.33—70.83	
	GH18P	74.00	78.00	73.10 ± 4.26	65.67—88.33	
	FAR16P	59.00	52.00	54.95 ± 5.23	48.00—70.00	0.77
	FAR17P	55.00	56.00	55.48 ± 2.83	52.00—63.00	
	FAR18P	57.00	57.00	55.69 ± 2.60	49.50—62.25	
	FAR18C	59.00	58.00	57.12 ± 3.17	49.33—68.67	
Plant height (PH) (inches)	GH18P	48.42	32.00	40.34 ± 6.28	27.00—57.00	0.67
	FAR18P	37.25	27.36	31.84 ± 3.15	26.00—40.75	
	FAR18C	35.50	26.95	31.07 ± 3.02	25.33—39.25	

*RILs, recombinant inbred lines; SD, standard deviation; H^2^, broad-sense heritability; FHB severity, mean of the symptomatic proportions of infected spikes; GH16P, GH17P, and GH18P represent the experiments conducted in greenhouse in 2016, 2017, and 2018, respectively, using the point-inoculation method. FAR16P, FAR17P, and FAR18P indicate the field experiments performed in 2016, 2017, and 2018, respectively, at the Fargo location with the point-inoculation method, while FAR18C was used to represent the corn-spawn inoculated experiment conducted at the Fargo location in 2018. DON content was assessed for grain samples of each line harvested from three FHB-inoculated experiments in 2018: GH18P, FAR18P, and FAR18C. The three DON testing experiments were designated as GH18P-DON, FAR18P-DON, and FAR18C-DON, respectively.*

On the other hand, FHB severity in the corn-spawn inoculated field experiment was relatively higher than in the point-inoculated field experiments. In addition, transgressive segregation was observed for both traits in the mapping population with higher and lower levels of FHB severity or DON accumulation than the parents (Figures 1, 2). However, the proportion of more resistant genotypes resulting from the transgressive segregation was low, ranging from 0.5 to 10% of the total number of RILs in different experiments. Only three RILs- WPDS070, WPDS111, and WPDS160- consistently showed better FHB resistance than the resistant parent across at least three experiments.

The mean DON accumulation in Surpresa and Wheaton varied significantly between greenhouse and field experiments, with the highest DON accumulation observed in the greenhouse experiment (Table 1). Among the field experiments, significantly higher DON accumulation occurred in the corn-spawn inoculated experiment than in the point-inoculated experiment. Surpresa accumulated DON in concentrations ranging from 3.4 parts per million (ppm) in FAR18P-DON to 10.3 ppm in FAR18C-DON. Wheaton, as expected, accumulated elevated DON levels ranging from 5.9 ppm in FAR18P-DON to 47.1 ppm in the GH18P-DON. Average DON accumulation in RILs followed the same order, with the highest accumulation observed in the greenhouse experiment and lowest in the point-inoculated field experiment. Mean DON accumulation in RILs varied between 0.3 and 202.4 ppm among the three experiments.

Analyses of variances showed significant genotype and genotype-by-environment interactions for both disease severity and DON accumulation across all experiments (*P* < 0.0001) (Table 2; data not shown for DON accumulation). The variances explained by environment and replication-by-environment were not significant (*P* > 0.05) (Table 2). Spearman’s correlation coefficient for disease severity ranged from –0.06 (*P* > 0.05) (FAR16P and FAR18C) to 0.49 (*P* < 0.0001) (FAR18P and FAR18C) across greenhouse and field experiments (Table 3). Correlation for DON accumulation levels observed across greenhouse and field experiments was very poor, ranging from –0.05 (*P* > 0.05) (FAR18P-DON and FAR18C-DON) to 0.14 (*P* < 0.05) (GH18P-DON and FAR18C-DON). Between disease severity and DON accumulation, however, the correlation ranged from –0.01 (FAR18P and FAR18P-DON) to 0.75 (*P* < 0.0001) (GH18P-DON and GH18P).

**TABLE 2:** Analysis of variance results for FHB severity measured across greenhouse and field environments in 187 Wheaton/Surpresa RILs.

Source	Greenhouse			Field		
	df	MS	*F*-value	df	MS	*F*-value
Year (Y)	2	587.21	0.39*^ns^*	2	1454.43	0.66*^ns^*
Rep × Year	6	523.94	0.35*^ns^*	4	22.73	0.01*^ns^*
Genotype	186	9926.77	6.62***	186	4484.51	2.03***
Genotype × Year	370	3194.35	2.13***	364	2895.00	1.31***

Year, Environment in which the analysis of variance is assessed; Rep, Biological replication; MS, Mean sum of squares. ***P < 0.0001; ^ns^P > 0.05.

**TABLE 3:** Spearman’s correlation coefficient between FHB disease severity (proportion of symptomatic spikelets) and deoxynivalenol (DON) levels calculated from individual experiments.

	GH16P	GH17P	GH18P	FAR16P	FAR17P	FAR18P	FAR18C	GH18P-DON	FAR18P-DON	FAR18C-DON
GH16P	…	0.35***	0.48***	0.28**	0.16*	0.26**	0.17*	0.36***	–0.08*^ns^*	0.00*^ns^*

GH17P	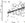	…	0.46***	0.26**	0.25**	0.18*	0.20*	0.46***	0.09*^ns^*	0.16*

GH18P	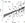	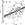	…	0.23*	0.27**	0.33***	0.32***	0.75***	0.10*^ns^*	–0.01*^ns^*

FAR16P	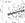	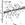	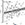	…	0.21**	0.09*^ns^*	0.06*^ns^*	0.11*^ns^*	–0.06*^ns^*	0.09*^ns^*

FAR17P	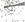	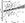	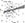	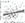	…	0.21*	0.08*^ns^*	0.06*^ns^*	0.17*	–0.17*

FAR18P	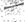	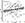	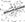	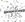	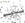	…	0.49***	0.17*	0.01*^ns^*	0.03*^ns^*

FAR18C	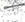	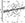	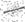	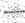	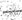	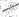	…	0.19*	0.18*	0.16*

GH18P-DON	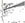	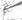	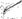	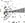	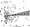	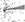	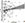	…	0.05*^ns^*	0.14*

FAR18P-DON	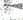	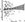	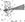	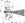	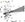	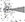	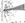	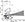	…	–0.05*^ns^*

FAR18C-DON	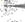	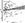	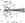	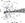	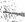	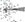	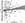	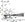	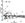	…

GH16P, GH17P, and GH18P represent the experiments conducted in greenhouse in 2016, 2017, and 2018, respectively, using the point-inoculation method. FAR16P, FAR17P, and FAR18P indicate the field experiments performed in 2016, 2017, and 2018, respectively, at the Fargo location with the point-inoculation method, while FAR18C was used to represent the corn-spawn inoculated experiment conducted at the Fargo location in 2018. DON content was assessed for grain samples of each line harvested from three FHB-inoculated experiments in 2018: GH18P, FAR18P, and FAR18C. The three DON testing experiments were designated as GH18P-DON, FAR18P-DON, and FAR18C-DON, respectively. ***P < 0.0001,**P < 0.001; *P < 0.05; ns, non-significant.

The distribution of plant height (PH) and days to anthesis (DA) among the RILs was continuous, indicating quantitative inheritance of the traits. The two parents differed significantly in DA, with Surpresa flowering 10.6 days, on average, later than Wheaton. Similarly, for PH, Wheaton, on average, was 13.5 inches shorter than Surpresa. Analysis of variance conducted to determine the sources of total variation observed in PH and DA showed significant contribution by the genotype, environment, and their interaction (data not shown).

Broad-sense heritability for disease severity was moderate, ranging from 0.47 for field experiments to 0.64 for greenhouse experiments, indicating that the assessment of FHB severity is reproducible (Table 1).

### Linkage Map Construction

The two-enzyme GBS approach identified a total of 5,681 SNPs with ≤ 30% missing data. Of the 5,681 SNP markers identified in the mapping population, 5,370 (94.53%) were mapped to 21 linkage groups, with at least 11 SNPs in each group, at a minimum threshold **LO**D value of 3 (Supplementary Figure 1 and Supplementary Table 1). Slightly over half of the SNP markers were mapped to the B genome (50.07%), followed by genome A (42.97%) and genome D (6.97%). The genetic linkage map spanned 3975.25 cM covering all 21 chromosomes of wheat with an average distance of 1.35 cM between each SNP marker (Supplementary Table 1).

### Quantitative Trait Loci for Fusarium Head Blight Resistance and Deoxynivalenol Accumulation

**FIGURE 4:**
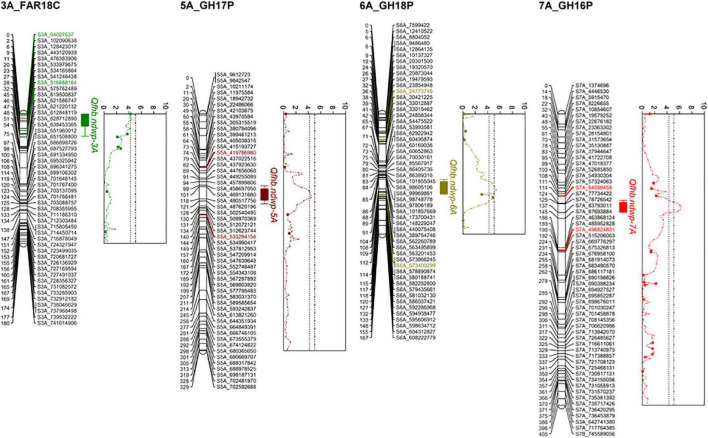
Linkage maps for chromosomes 3A, 5A, 6A, and 7A showing the respective QTL for type II FHB resistance detected in the Wheaton/Surpresa RIL population. The positions of marker loci are shown on the right and the centimorgan (cM) distances between the loci are shown on the left of the linkage groups.

**FIGURE 5:**
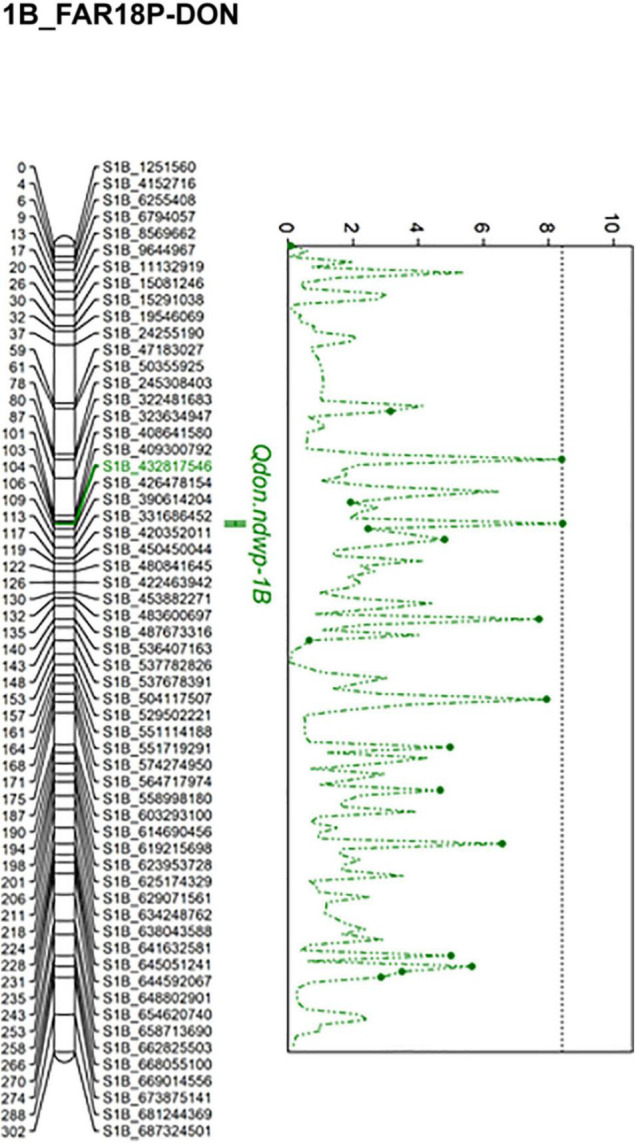
Linkage map for chromosome 1B showing the QTL for resistance to DON accumulation (type III FHB resistance) detected in the Wheaton/Surpresa RIL population. The positions of marker loci are shown on the right and the centimorgan (cM) distances between the loci are shown on the left of the linkage groups.

Composite interval mapping (CIM) detected four significant QTL (*Qfhb.ndwp-3A*, *Qfhb.ndwp-5A*, *Qfhb.ndwp-6A*, and *Qfhb.ndwp-7A*) for FHB type II resistance on chromosomes 3A, 5A, 6A, and 7A, respectively (Table 4 and Figure 4). *Qfhb.ndwp-5A* and *Qfhb.ndwp-6A* were derived from the resistant parent Surpresa while *Qfhb.ndwp-3A* and *Qfhb.ndwp-7A* were derived from the susceptible parent Wheaton. Besides QTL for FHB resistance, a QTL (*Qdon.ndwp-1B*) for resistance to DON accumulation (type III resistance) was detected on chromosome 1B (Figure 5). *Qdon.ndwp-1B* was derived from the susceptible parent Wheaton. The QTL, their positions, the experiment in which the QTL were detected are presented in Table 4. Comparisons of these QTL with previously reported QTL on the same chromosomes or genomic regions are summarized on Supplementary Table 2.

**TABLE 4:** Summary of QTL detected for FHB severity (type II resistance) and DON accumulation (type III resistance) by composite interval mapping (CIM) in the Wheaton/Surpresa RIL population.

Type	QTL	Chr	EXP	Flanking SNP markers	LOD	*R* ^2^	Add.	Parent
SEV	*Qfhb.ndwp-3A*	3A	FAR18C	S3A_64027637—S3A_516888164	4.5*	0.10	–3.09	WHTN
SEV	*Qfhb.ndwp-5A*	5A	GH17P	S5A_419786980—S5A_533294156	4.48*	0.11	5.53	SPRS
SEV	*Qfhb.ndwp-6A*	6A	GH18P	S6A_24773746—S6A_573400299	5.42**	0.13	5.53	SPRS
SEV	*Qfhb.ndwp-7A*	7A	GH16P	S7A_64598458—S7A_496824831	6.3*	0.14	–7.55	WHTN
DON	*Qdon.ndwp-1B*	1B	FAR18P-DON	S1B_432817546	8.44*	0.19	–0.81	WHTN

QTL, quantitative trait loci; SEV, disease severity; DON, deoxynivalenol; Chr, chromosome; EXP, experiment in which the QTL was detected; GH16P, GH17P, and GH18P represent the experiments conducted in greenhouse in 2016, 2017, and 2018, respectively, using the point-inoculation method. FAR18P indicates the field experiment performed in 2018 with the point-inoculation method, while FAR18C was used to represent the corn-spawn inoculated experiment conducted at the Fargo location in 2018. FAR18P-DON represents DON testing for the grain samples harvested from field experiment FAR18P. LOD, logarithm of odds; *P < 0.05; **P < 0.01 following 1,000 permutation tests; R^2^, proportion of phenotypic variance explained by each QTL; Add., additive effect denoting the contribution of resistant or susceptible allele; WHTN, Wheaton; SPRS, Surpresa.

*Qfhb.ndwp-5A* was detected in the GH17P experiment and was mapped to a 66 cM genetic distance between SNPs S5A_419786980 and S5A_5332941. *Qfhb.ndwp-5A* explained 11% of the total phenotypic variation in disease severity.

*Qfhb.ndwp-6A* was identified in the GH18P experiment, which spanned 76 cM between flanking SNP markers S6A_24773746 and S6A_573400299 explaining 13% of the total phenotypic variation in disease severity.

*Qfhb.ndwp-3A* was detected in the FAR18C experiment and mapped to 28 cM genetic distance between SNPs S3A_64027637 and S3A_516888164 explaining 10.4% of the total phenotypic variation in disease severity.

*Qfhb.ndwp-7A* was identified in the GH16P experiment, which had the largest effect among the QTL detected for FHB resistance and explained 14.4% of the total phenotypic variation in disease severity. This QTL was delineated to a 76 cM interval between SNPs S7A_64598458 and S7A_496824831.

The QTL for resistance to DON accumulation on chromosome 1B, *Qdon.ndwp-1B*, was detected in the FAR18P-DON experiment. *Qdon.ndwp-1B* explained 19% of the total phenotypic variation in DON accumulation by the RILs. Several peaks were detected on chromosome 1B based on CIM mapping, however, only one SNP marker (S1B_432817546) associated with the QTL was found to be significant after 1,000 permutation tests.

### Quantitative Trait Loci for Days to Anthesis and Plant Height

QTL analyses using the DA and PH data from individual environments led to the detection of a total of 2 QTL for PH and 5 QTL for DA (Table 5 and Supplementary Figures 2–6).

**TABLE 5:** Summary of QTL detected for agronomic traits by composite interval mapping (CIM) in the Wheaton/Surpresa RIL population.

Type	QTL	Chr	EXP	Flanking SNP markers	LOD	*R* ^2^	Add.
DA	*Qda.ndwp-2B*	2B	GH17P	S2B_53430011—S2B_89567835	5.72**	0.13	–2.02
DA	*Qda.ndwp-2B*	2B	FAR17P	S2B_53430011—S2B_89567835	8.63**	0.19	–1.26
DA	*Qda.ndwp-2B*	2B	GH18P	S2B_53430011—S2B_89567835	5.45*	0.13	–1.53
DA	*Qda.ndwp-2B*	2B	FAR18C	S2B_53430011—S2B_89567835	5.12*	0.12	–1.11
DA	*Qda.ndwp-2D*	2D	FAR17P	S2D_11522287	12.47**	0.26	–1.66
DA	*Qda.ndwp-2D*	2D	GH18P	S2D_11522287	8.91**	0.20	–1.91
DA	*Qda.ndwp-2D*	2D	FAR18P	S2D_11522287	12.74**	0.27	–1.55
DA	*Qda.ndwp-2D*	2D	FAR18C	S2D_11522287	10.34**	0.23	–1.50
DA	*Qda.ndwp-5A*	5A	FAR18P	S5A_552675375	6.28**	0.14	1.24
DA	*Qda.ndwp-6B*	6B	GH16P	S6B_481863585	5.59*	0.13	0.34
DA	*Qda.ndwp-7A*	7A	GH18P	S7A_51100870—S7A_64598458	7.20**	0.16	1.57
PH	*Qph.ndwp-2D*	2D	FAR18P	S2D_11522287	5.15**	0.12	–0.95
PH	*Qph.ndwp-2D*	2D	FAR18C	S2D_11522287	6.70**	0.15	–1.04
PH	*Qph.ndwp-4D*	4D	FAR18P	S4D_43140163—S4D_45526446	9.04**	0.20	–1.63
PH	*Qph.ndwp-4D*	4D	FAR18C	S4D_43140163—S4D_45526446	8.19**	0.18	–1.50

QTL, quantitative trait loci; DA, days to anthesis; PH, plant height; Chr, chromosome; EXP, experiment in which the QTL was detected; GH16P, GH17P, and GH18P represent the experiments conducted in greenhouse in 2016, 2017, and 2018, respectively, using the point-inoculation method. FAR16P, FAR17P, and FAR18P indicate the field experiments performed in 2016, 2017, and 2018, respectively, at the Fargo location with the point-inoculation method, while FAR18C was used to represent the corn-spawn inoculated experiment conducted at the Fargo location in 2018. LOD, logarithm of odds; *P < 0.05; **P < 0.01 following 1,000 permutation tests; R^2^, proportion of phenotypic variance explained by each QTL; Add., additive effect denoting the contribution of resistant or susceptible allele.

Of the 5 QTL detected for DA in this study, QTL detected on chromosome 2B (*Qda.ndwp-2B*) and 2D (*Qda.ndwp-2D*) (Supplementary Figures 2, 3), explaining between 12 and 27% of the total phenotypic variation in DA, respectively, and were stable and expressed consistently across multiple environments (Table 5). Three minor QTL detected on chromosomes 5A (*Qda.ndwp-5A*), 6B (*Qda.ndwp-6B*) and 7A (*Qda.ndwp-7A*) explained 13 to 16% of the total phenotypic variation in DA, respectively (Table 5 and Supplementary Figure 4). It is worth to note that *Qda.ndwp-7A* was localized at the genomic region closely linked to where the FHB resistance QTL, *Qfhb.ndwp-7A*, was mapped (Figure 4; and Supplementary Figure 4).

The two QTL detected for PH were localized on chromosome 2D (*Qph.ndwp-2D*) and 4D (*Qph.ndwp-4D*) (Supplementary Figures 5, 6), which showed consistency across multiple environments and explained 12 and 20% of the total phenotypic variation, respectively (Table 5).

## Discussion

### Fusarium Head Blight and Trait Correlations

In this study, we evaluated FHB-related traits, FHB severity and DON accumulation, in both greenhouse and field experiments. Higher disease severity and DON accumulation were observed in the greenhouse experiments than in the field experiments (Table 1). Correlations for disease severity and DON accumulation across all greenhouse experiments were higher than those across field experiments. This may be due to the controlled environment in the greenhouse that offered conducive conditions for disease development, higher disease severity and DON accumulation levels, and higher correlations between the two FHB-related traits. Correlations between FHB severity and DON accumulation have been extensively studied; however, the results varied among different experiments (Ma et al., 2006). In our study, a strong positive correlation was observed between FHB severity and DON accumulation in greenhouse experiments, while the results on correlations between the two FHB-related traits were inconclusive in the field experiments (Table 3), suggesting a strong influence of the environment conditions in field experiments.

Besides FHB-related traits such as FHB severity and DON accumulation, morphological and phenological traits—mainly plant height (PH), anther extrusion, and days to anthesis—may be involved in FHB development. Several studies have demonstrated an inverse relationship between PH and FHB severity, with shorter stature genotypes developing more severe FHB symptoms (Mesterházy, 1995; Buerstmayr et al., 2000; Gervais et al., 2003). This inverse relationship could be explained under two possible circumstances; i. association between the genomic loci conferring PH and the FHB resistance loci, or ii. the effect of microclimate on disease development, or both (Yan et al., 2011). In this study, despite variation in PH among the RILs, no significant association was detected between PH and FHB resistance (data not shown). Furthermore, the QTL identified for PH are different from those for FHB resistance in the mapping population (Tables 4, 5). This suggests that FHB resistance is independent of PH in this mapping population.

Several genes known to regulate flowering and heading time such as the vernalization requirement genes *Vrn-A1* (5AL), *Vrn-B1* (5B), the earliness *per se* (*Eps*) loci, and the photoperiod insensitivity gene *Ppd-D1a* (2DS) have been associated with FHB resistance (Buerstmayr et al., 2020). In the present study, no systematic associations were observed between DA and FHB severity across the different environments, and among the four QTL for the type II FHB resistance, only *Qfhb.ndwp-7A* was showed to be closely linked to the QTL for DA (*Qda.ndwp-*7A) on chromosome 7A. Therefore, positive or negative correlations previously reported by different studies for flowering time and FHB resistance are most likely due to the effect of weather conditions during inoculation time (Buerstmayr et al., 2009, 2020).

### Fusarium Head Blight Resistance Quantitative Trait Loci in the Brazilian Cultivar Surpresa

Two FHB resistance QTL derived from the resistant parent Surpresa were identified in the mapping population, including *Qfhb.ndwp-5A* and *Qfhb.ndwp-6A*.

Based on a study that integrated 716 FHB type II and III resistance QTL detected from 113 mapping experiments published in the past two decades (Zheng et al., 2021), at least 60 QTL for type II resistance to FHB have been identified in chromosome 5A originating from wheat cultivars world-wide, including Frontana. Frontana harbors two type II FHB resistance QTL on chromosome 5A spanning physical intervals between 205 and 524 Mb, including the physical location of the *Qfhb.ndwp-5A* QTL detected in this study (Supplementary Table 2). This indicates the possibility that the FHB resistance QTL on chromosome 5A in Frontana and Surpresa may be the same or tightly linked together. Zheng et al. (2021) refined the QTL detected on chromosome 5A by removing QTL with > 20 Mb physical interval and those reported in less than five studies, leaving 5 high confidence QTL. A total of 58 differentially expressed genes were identified in the physical interval spanning *Qfhb.ndwp-5A*. Further analysis of the differentially expressed genes led to a putative candidate gene *TraesCS5A02G26400* encoding 2-oxoglutarate (2OG) and Fe (II)-dependent oxygenase that was upregulated during *Fusarium graminearum* infection based on published transcriptomic and proteomic data. However, it is remained to be known if *TraesCS5A02G26400* confers FHB resistance.

Zheng et al. (2021) reported 19 QTL for type II FHB resistance on chromosome 6A from various wheat cultivars, including Frontana. Frontana harbors a type II FHB resistance on chromosome 6A spanning 3 cM genetic distance between markers WPT-7204 and WPT-744786 (physical location: 610–617 Mb) (Supplementary Table 2). Based on the physical location of *Qfhb.ndwp-6A* (physical location: 24–573 Mb), it appears that *Qfhb.ndwp-6A* is different from the one originating from Frontana. Furthermore, Zheng et al. (2021) identified four high confidence meta QTL (*hcmQTL 57—60*) on chromosome 6A spanning the physical interval of *Qfhb.ndwp-6A* with 116 differentially expressed genes. Combined analysis of differentially expressed genes and differentially accumulated proteins led to the identification of a potential candidate gene *TraesCS6A02G059600* for *Qfhb.ndwp-6A*. *TraesCS6A02G059600* was found to encode Glutathione S-transferase (GST) gene which was upregulated during *Fusarium graminearum* infection. GSTs have multiple functions such as detoxification, isomerization, and peroxidation, and have been shown to be involved in responses of plants to biotic and abiotic stresses (Wahibah et al., 2018). Recently, Wang et al. (2020) have cloned the *Fhb7* gene, which encodes a GST conferring FHB resistance by detoxifying trichothecenes through de-epoxidation. The GST gene was gained through horizontal gene transfer (HGT) from an endophytic Epichloë species (Wang et al., 2020). It would be interesting to know if *TraesCS6A02G059600* has the same function as *Fhb7*.

### Fusarium Head Blight Resistance Quantitative Trait Loci in the Susceptible Cultivar Wheaton

Three QTL for resistance to FHB (*Qfhb.ndwp-7A* and *Qfhb.ndwp-3A*) or DON accumulation (*Qdon.ndwp-1B*) derived from Wheaton were detected in this study. Cai et al. (2016) also identified two QTL on chromosome 2B for type II resistance derived from Wheaton in a RIL mapping from a cross between Wheaton and Haiyanzhong (HYZ), a Chinese wheat landrace with a high level of resistance to FHB,. These results indicate that minor effect FHB resistance QTL exist even in a susceptible wheat genotypes like Wheaton.

*Qfhb.ndwp-7A* explained the largest phenotypic variation (14%) for FHB resistance in the NIL population. However, this QTL is closely linked to a *Qda.ndwp-7A*, conferring the DA phenotype. Early or late flowering lines often escape the high inoculum pressure and develop low levels of FHB severity depending on the environments. In this study, Wheaton underwent anthesis 14 days later than 58% of the lines evaluated in the GH16P experiment. Therefore, FHB resistance conferred by *Qfhb.ndwp-7A* may be due to the late flowering phenotype associated with *Qda.ndwp-7A*. Based on meta-QTL analysis by Zheng et al. (2021), at least 25 type II FHB resistance QTL have been mapped on chromosome 7A (Zhou et al., 2004; Jia et al., 2005; Mardi et al., 2006; Semagn et al., 2007; Yu et al., 2008; Jayatilake et al., 2011). Jayatilake et al. (2011) detected a major QTL on chromosome 7A, *Fhb7AC*, of Sumai3 origin. *Fhb7AC* spans between Xbarc174 (physical position: 116002885) and Xwmc9 (physical position: 394904915) encompassing the *Qfhb.ndwp-7A* QTL (Supplementary Table 2), indicating the likelihood of these two QTL being localized at the same genomic region or closely linked.

*Qfhb.ndwp-3A* on chromosome 3A was identified in the FAR18C experiment and explained 10% of the total phenotypic variation in FHB severity. Several QTL for type II FHB resistance have been detected on chromosome 3A (Mardi et al., 2006; Zhang et al., 2012; Cai and Bai, 2014). The QTL detected on chromosome 3A derived from a Chinese wheat landrace Huangcandou (HCD), flanked by Xcfa2134 and Xgwm2 (physical position: 60–509 Mb) (Supplementary Table 2), encompasses the physical location of *Qfhb.ndwp-3A* and these two QTL are likely the same one or allelic (Cai and Bai, 2014).

*Qdon.ndwp-1B* was detected in the FAR18C-DON experiment and explained 19% of the total phenotypic variation in DON accumulation among RILs. Several studies have identified type III resistance QTL on chromosome 1B (Yu et al., 2008; Ágnes et al., 2014; Petersen et al., 2017). The QTL positioned closest to *Qdon.ndwp-1B* was detected in a US winter wheat cultivar NC-Neuse, flanked by markers IWA6290 and WMC419 (peak location: 310 Mb), and is approximately 120 Mb proximal from the peak marker S1B_432817546 detected in this study (Supplementary Table 2). This indicates that *Qdon.ndwp-1B* is likely novel.

### Frontana vs. Surpresa

Frontana and Surpresa are both Brazilian spring wheat cultivars and share the Brazilian landraces Polyssu and Alfredo Chaves in their pedigrees (van Beuningen and Busch, 1997). Frontana was a widely used FHB resistance source in the Brazilian, North American, and Canadian wheat breeding programs prior to the introduction of Asian germplasms (Zhu et al., 2019). FHB resistance in Frontana has been characterized and validated across multiple studies and is primarily conferred by QTL on chromosome 3A (Steiner et al., 2004; Mardi et al., 2006; Berzonsky et al., 2007; Yabwalo et al., 2011; Szabo-Hever et al., 2018). Besides the QTL on chromosome 3A, QTL from numerous chromosomes including 4A, 5A, 6A, 6B, 7A, 4D, some of which are coincident with QTL for PH and DA, contribute to FHB resistance in Frontana (Steiner et al., 2004; Mardi et al., 2006; Berzonsky et al., 2007; Szabo-Hever et al., 2018). Based on the QTL detected from Surpresa in this study, Frontana and Surpresa share the genomic interval contributing to the type II FHB resistance on chromosome 5A, potentially indicating they have the same candidate gene identified by Zheng et al. (2021) contributing to the resistance. However, the QTL on chromosome 6A identified in this study, *Qfhb.ndwp-6A*, is different from the one identified in Frontana, indicating that Frontana and Surpresa do not seem to share the QTL for FHB resistance originating from chromosome 6A.

### Transgressive Segregants

In this study, three RILs (WPDS070, WPDS111, and WPDS160) were identified as transgressive segregants exhibiting better FHB resistance than the resistant parent Surpresa across multiple experiments. These RILs may contain FHB resistance QTL derived from both parents (Surpresa and Wheaton). However, a combination of favorable marker alleles for the FHB resistant QTL were not detected in these transgressive segregants. For example, WPDS 160, a transgressive segregant that consistently showed better FHB resistance than the resistant parent, did not carry any favorable marker alleles associated with the QTL detected in this study. This may be due to the favorable marker alleles not closely enough linked to the FHB resistant loci, leading to recombination between the markers and the QTL. The interactions between genes derived from the parental wheat genotypes might also contribute to the better resistance of the NILs than their resistant parent.

## Conclusion

In summary, we observed that the type II resistance conferred by Surpresa appeared not to be consistent across different environments and the two QTL detected from Surpresa had a minor effect on FHB resistance. Interestingly, minor effect QTL for FHB resistance were also identified in Wheaton, the susceptible wheat parent used in the mapping population. Furthermore, some NILs derived from the cross between Wheaton and Surpressa exhibited a better FHB resistance than the resistant parent, indicating that FHB resistance can be improved by pyramiding minor QTL with additive effect. To use these minor QTL for FHB resistance improvement in wheat breeding programs, it is essential to develop effective DNA markers for their selection and combination.

## Data Availability Statement

The datasets presented in this study can be found in online repositories. The names of the repository/repositories and accession number(s) can be found in the article/Supplementary Material.

## Author Contributions

SZ conceived and designed the study. KP, BP, and SZ developed the RIL mapping population. BP, JM, YLe, AK, and SZ carried out the phenotyping for the mapping population. YLi, JH, and XL generated the genotype data. BP, YLi, YLe, XL, and SZ conducted the data analysis. BP and SZ wrote the manuscript draft. All authors reviewed, edited, and approved the final version of the manuscript.

## Author Disclaimer

Any opinions, findings, conclusions, or recommendations expressed in this publication are those of the authors and do not necessarily reflect the view of the U.S. Department of Agriculture.

## Conflict of Interest

The authors declare that the research was conducted in the absence of any commercial or financial relationships that could be construed as a potential conflict of interest.

## Publisher’s Note

All claims expressed in this article are solely those of the authors and do not necessarily represent those of their affiliated organizations, or those of the publisher, the editors and the reviewers. Any product that may be evaluated in this article, or claim that may be made by its manufacturer, is not guaranteed or endorsed by the publisher.
