# Supplementary material for: Molecular Mapping of Quantitative Trait Loci for Fusarium Head Blight Resistance in the Brazilian Spring Wheat Cultivar “Surpresa”
Source: Front Plant Sci. 2022 Jan 24;12:778472. doi: 10.3389/fpls.2021.778472 (PMC8818699; doi:10.3389/fpls.2021.778472)
Supplement: Supplementary file 1 [file Data_Sheet_1.PDF]

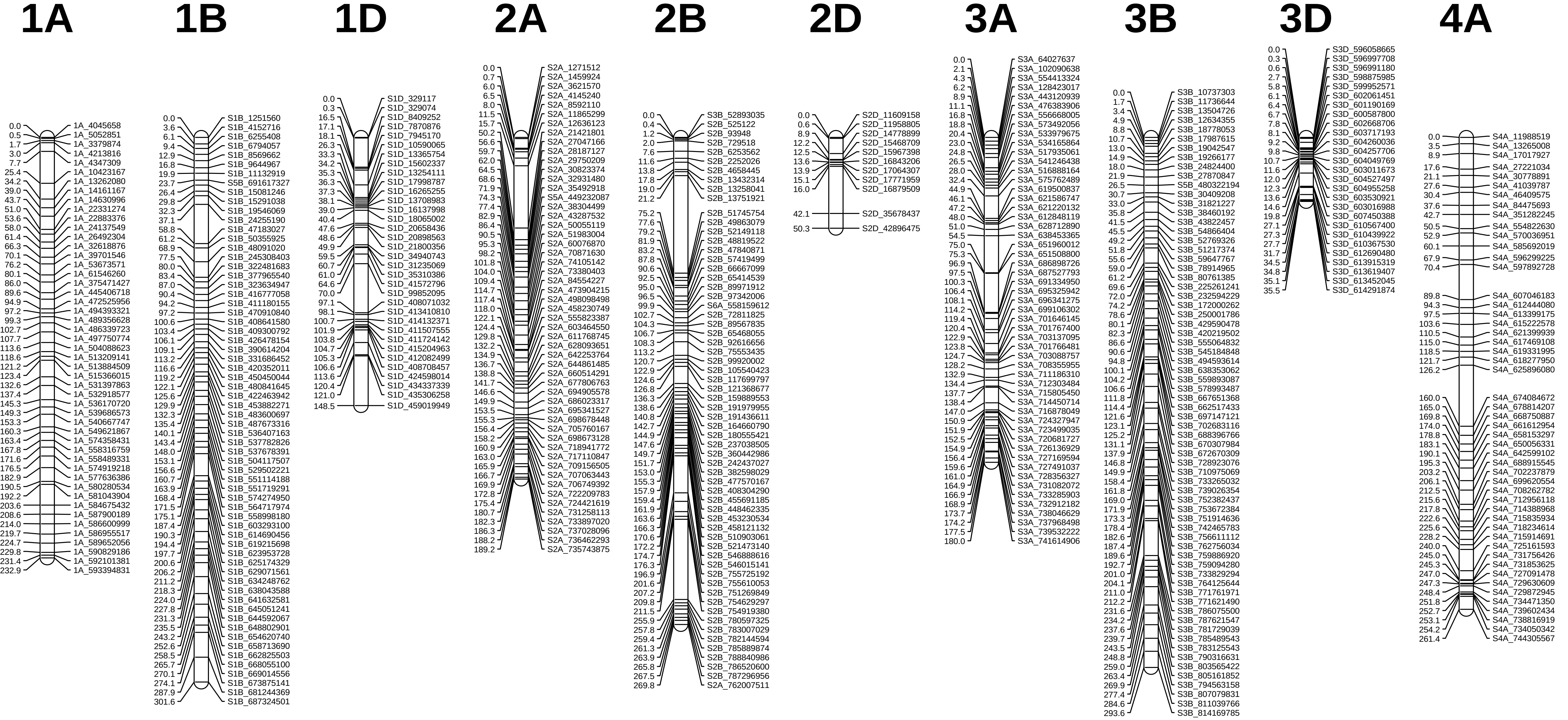

Supplementary Figure 1. Genetic linkage maps constructed in the Wheaton/Surpresa derived population.

The position of marker loci is shown to the right of the linkage groups, and centimorgan (cM) distances between loci are shown to the left.

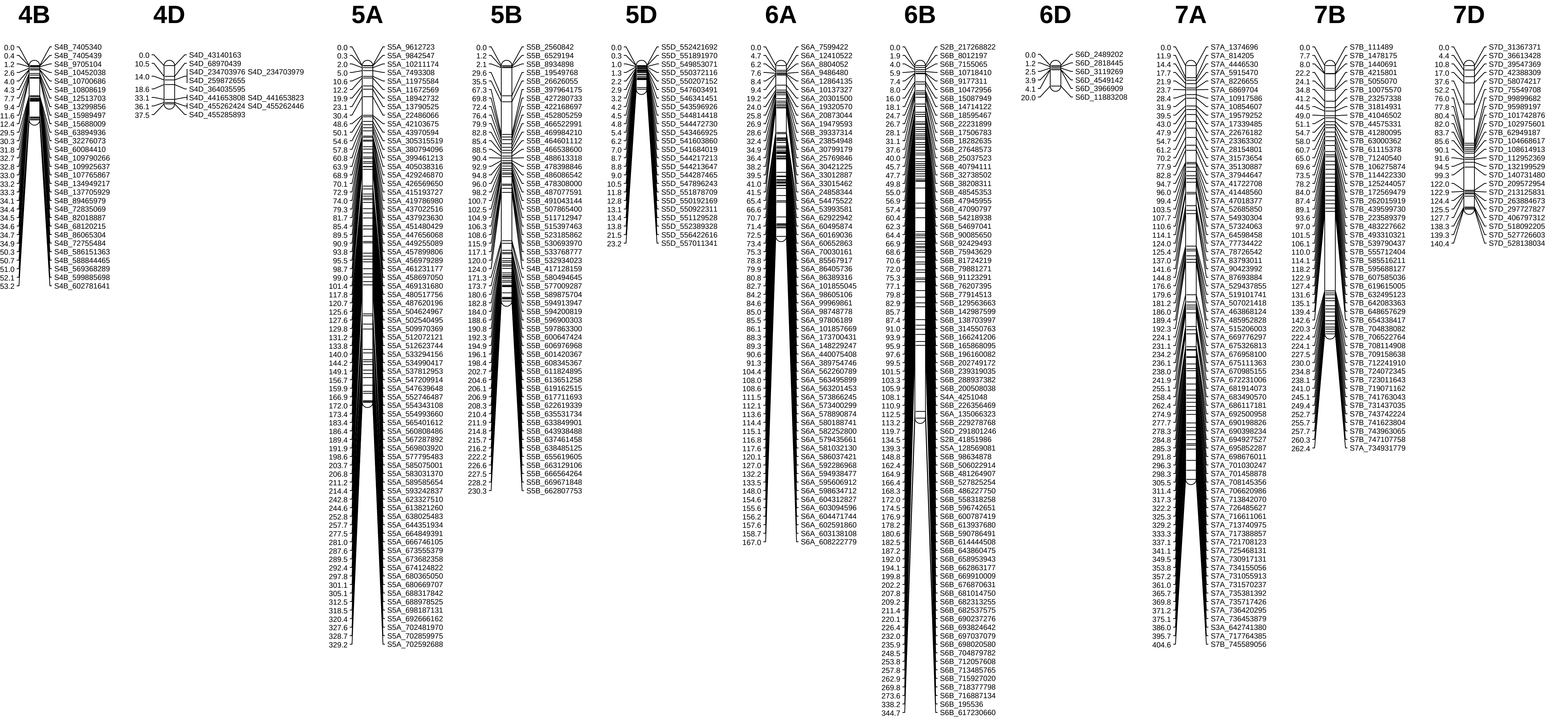

Supplementary Figure 1. Genetic linkage maps constructed in the Wheaton/Surpresa derived population (continued).

The position of marker loci is shown to the right of the linkage groups, and centimorgan (cM) distances between loci are shown to the left.

QTL for Days to Anthesis: Wheaton x Surpresa RIL

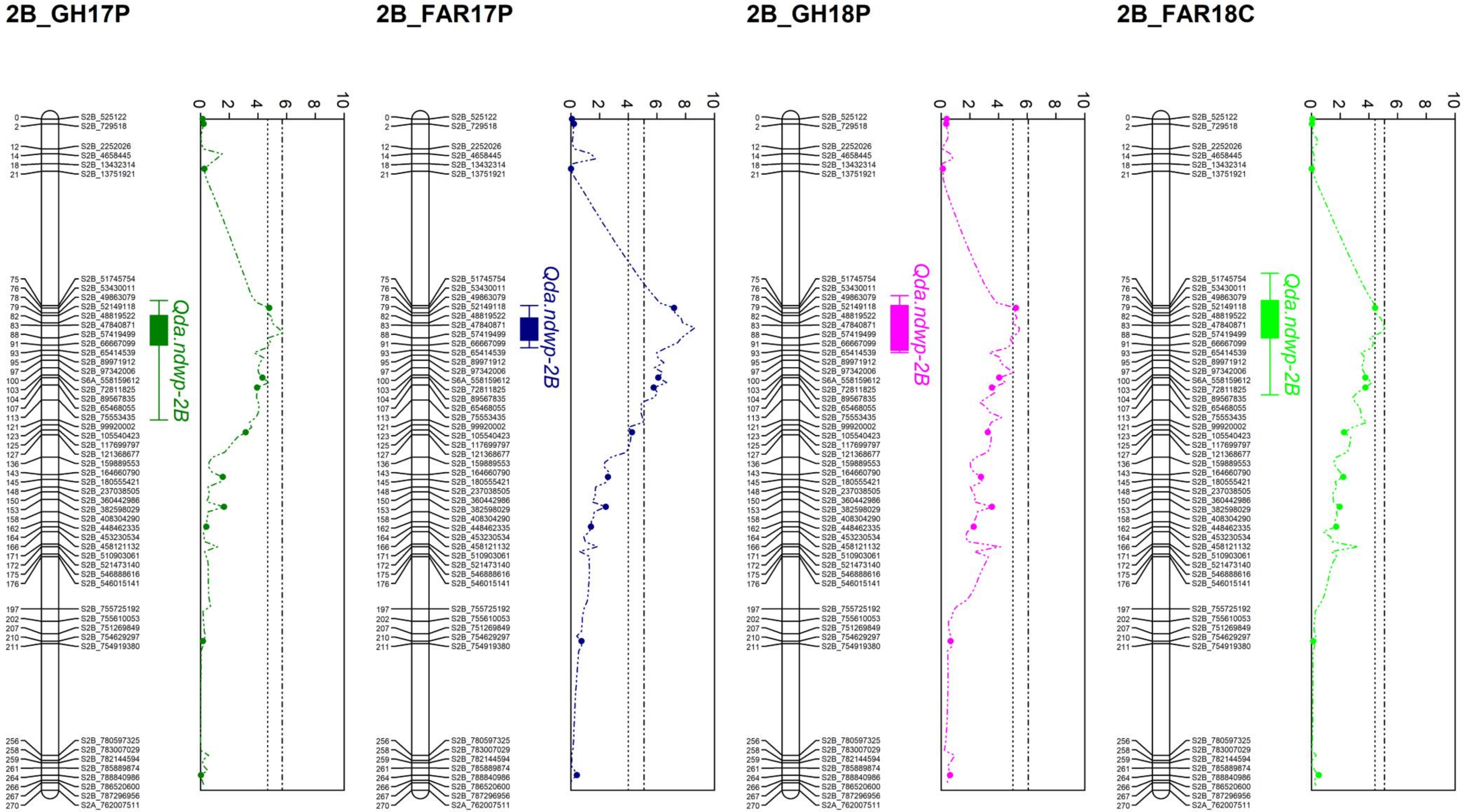

Supplementary Figure 2. Linkage maps for chromosome 2B showing the QTL for days to anthesis derived from the Wheaton/Surpresa RIL population.

The position of marker loci ish shown to the right of the linkage groups, and centimorgan (cM) distances between loci are shown to the left.

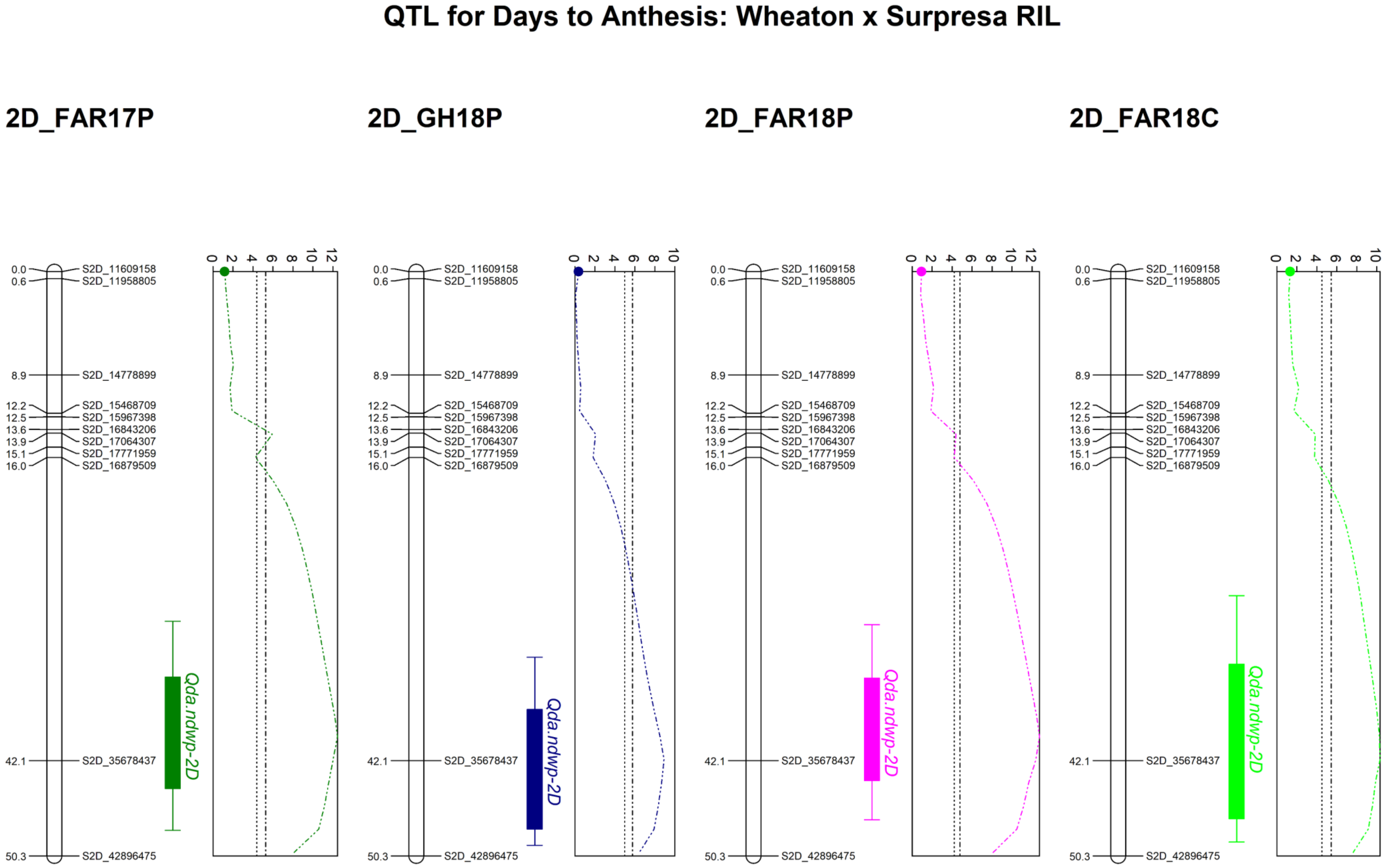

Supplementary Figure 3. Linkage maps for chromosome 2D showing the QTL for days to anthesis derived from the Wheaton/Surpresa RIL population.

The position of marker loci is shown to the right of the linkage groups, and centimorgan (cM) distances between the loci are shown to the left.

QTL for Days to Anthesis: Wheaton x Surpresa RIL

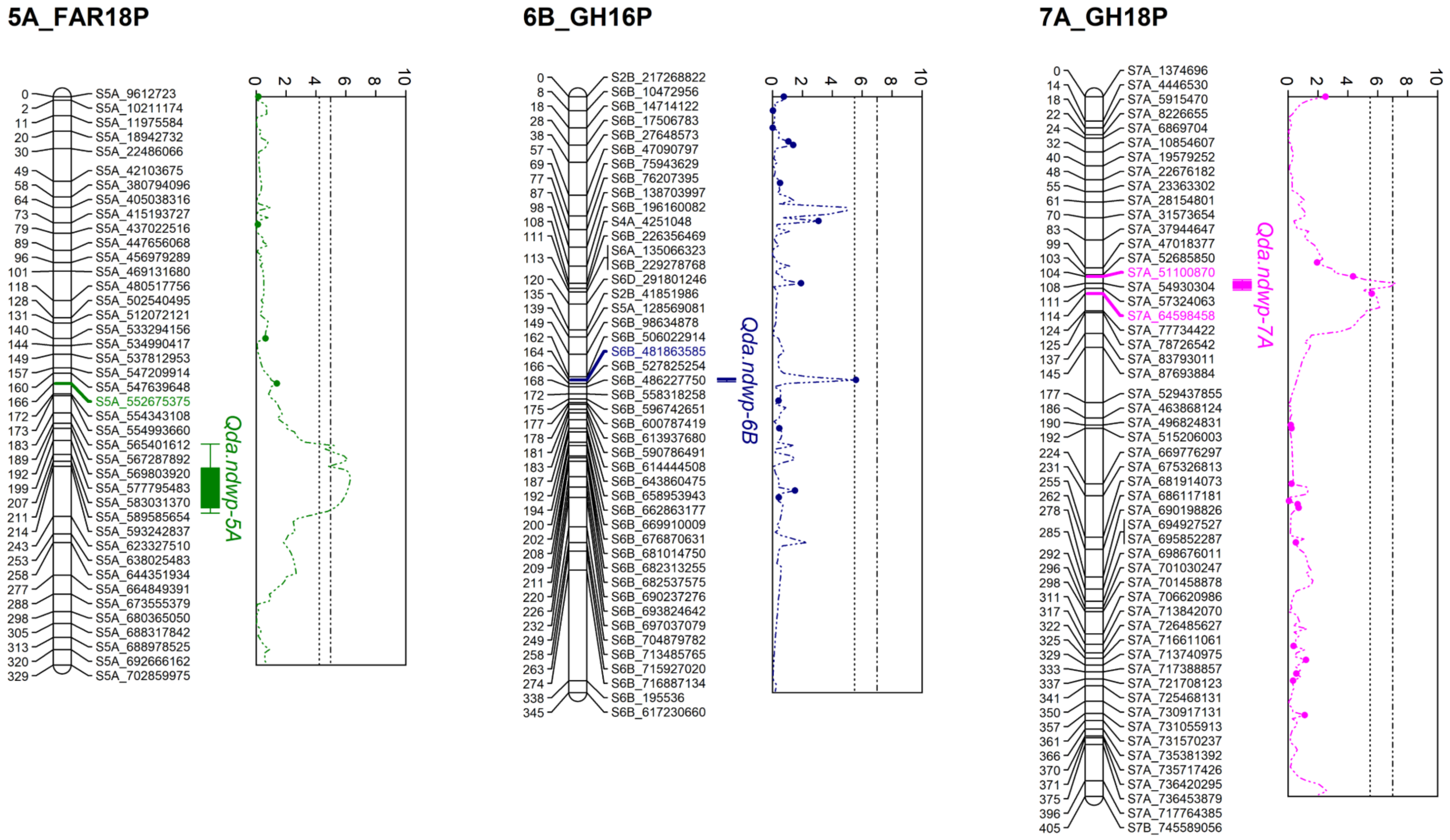

Supplementary Figure 4. Linkage maps for chromosomes 5A, 6B, and 7A showing respective QTL for days to anthesis derived from the Wheaton/Surpresa RIL population. The position of marker loci is shown to the right of the linkage groups, and centimorgan (cM) distances between the loci are shown to the left.

QTL for Plant Height: Wheaton x Surpresa RIL

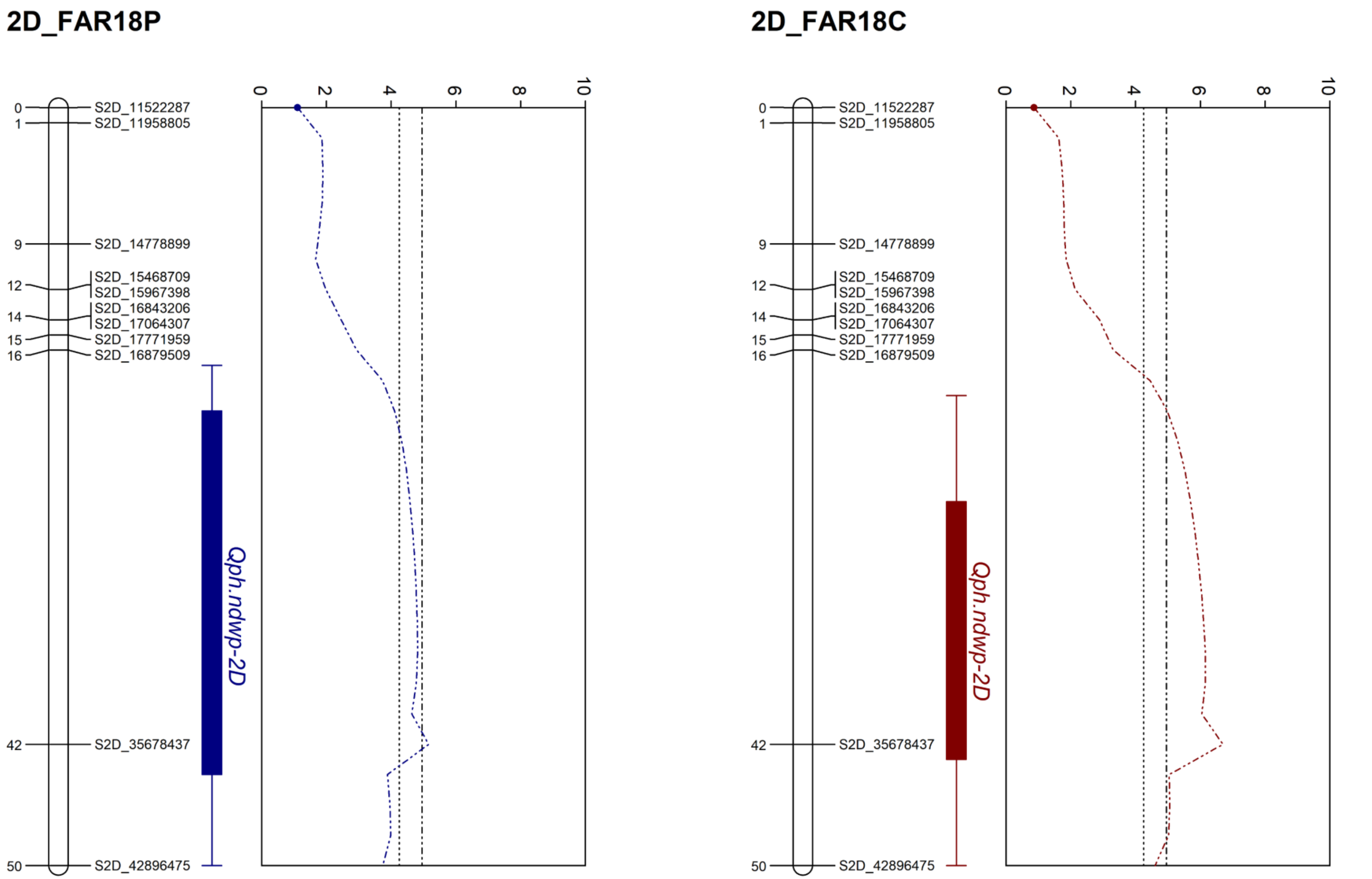

Supplementary Figure 5. Linkage maps for chromosome 2D showing the QTL for plant height derived from the Wheaton/Surpresa RIL population.

The position of marker loci is shown to the right of the linkage groups, and centimorgan (cM) distances between the loci are shown to the left.

QTL for Plant Height: Wheaton x Surpresa RIL

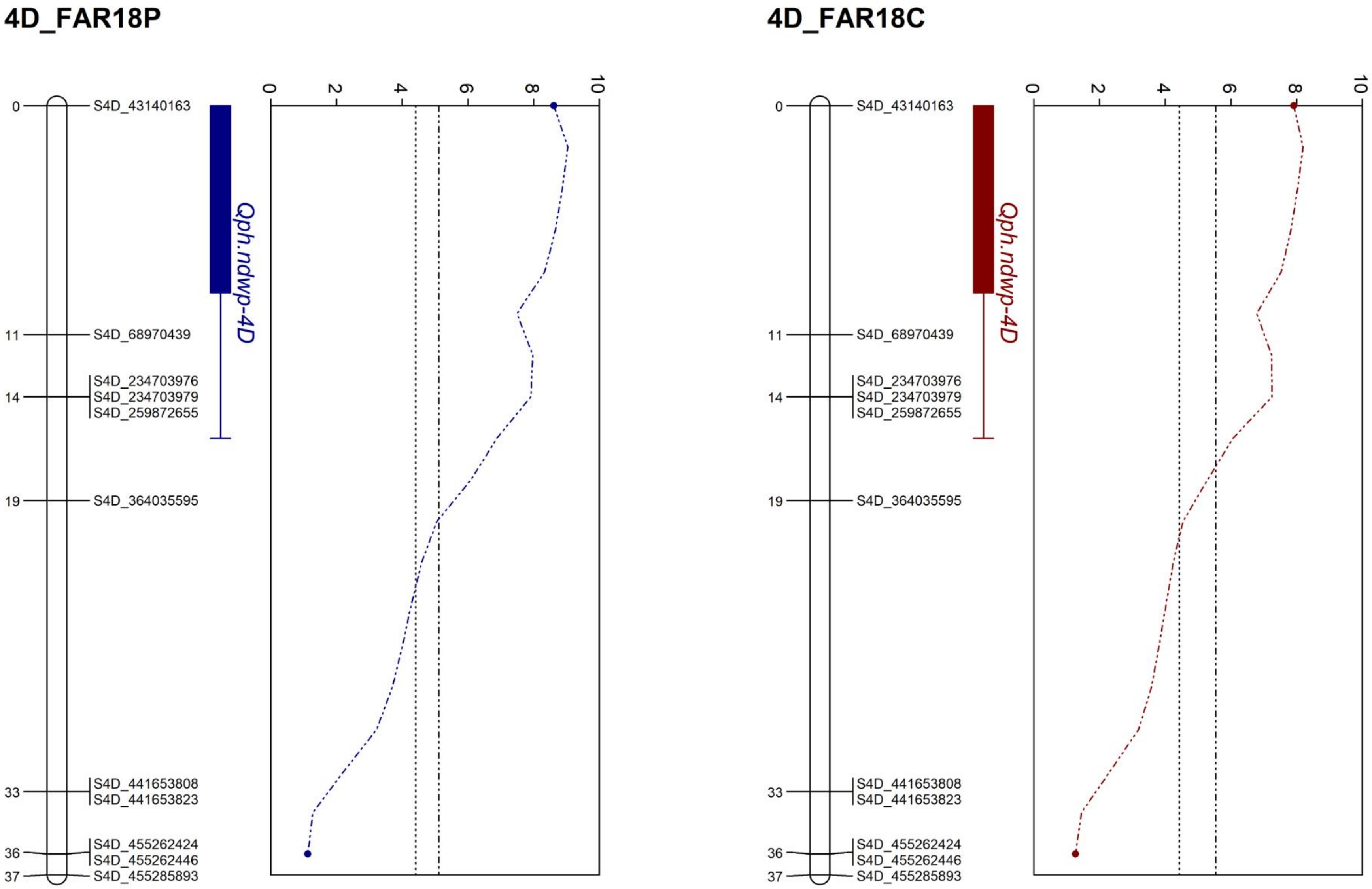

Supplementary Figure 6. Linkage maps for chromosome 4D showing the QTL for plant height derived from the Wheaton/Surpresa RIL population.

The position of marker loci is shown to the right of the linkage groups, and centimorgan (cM) distances between the loci are shown to the left.
